# Influences of sterol regulatory element binding protein-1c silencing on glucose production in HepG2 cells treated with free fatty acid

**DOI:** 10.1186/s12944-019-1026-3

**Published:** 2019-04-06

**Authors:** Xiu-Ping Bai, Feng Dong, Guo-Hua Yang, Lei Zhang

**Affiliations:** 1 Endocrinology Division, The Second Hospital of ShanXi Medical University, TaiYuan, 030001 ShanXi China; 2 Diabetes Division, University of Texas Health Science Center, San Antonio, TX USA; 3 Central Laboratory, The Second Hospital of ShanXi Medical University, TaiYuan, 030001 ShanXi China

**Keywords:** Glucose production, SREBP-1c silencing, Free fatty acid, HepG2 cells

## Abstract

**Background:**

Elevation of exogenous free fatty acid (FFA) level leads to insulin resistance (IR) in liver, IR is manifested by elevated hepatic glucose production. We aim to study whether inhibition of endogenous fatty acid synthesis could decrease hepatic glucose production.

**Methods:**

Low-passage HepG2 cells derived from human liver tissue were cultured in medium supplemented with FFA to induce IR, the influences of sterol regulatory element binding protein-1c (SREBP-1c) silencing on glucose production of HepG2 cells were investigated, and genes responsible for fatty acid and glucose metabolism were detected by real-time PCR.

**Results:**

Compared with HepG2 cells cultured in normal growth medium, glucose production of HepG2 cells treated by FFA was significantly increased {[(0.28 ± 0.01) vs (0.83 ± 0.02)] umol.ug^− 1^ protein, *n* = 6 wells, *P* < 0.01}; the mRNA expression of phosphoenolpyruvate carboxylase kinase (PEPCK) and glucose-6-phosphatase (G6PC) in HepG2 cells increased by more than 5-fold and 3-fold, respectively; the mRNA expression of fatty acid synthase (FAS) and stearoyl-CoA desaturase-1 (SCD1) increased by approximately 4-fold and 1.1-fold, respectively; the mRNA expression of carnitine palmitoyltransferase-1 (CPT-1) changed slightly. Compared with the scrambled siRNA control, glucose production of HepG2 cells treated by FFA significantly increased after SREBP-1c silencing {[(0.018 ± 0.001) vs (0.028 ± 0.002)] umol.ug^− 1^ protein, *n* = 6 wells, *P* < 0.01}; the mRNA expression of PEPCK and G6PC increased by approximately 1.5-fold and 5-fold, respectively, but the mRNA expression of FAS, SCD1 and CPT-1 changed slightly.

**Conclusions:**

SREBP-1c silencing further augmented glucose production of HepG2 cells treated by FFA significantly, genes responsible for fatty acid synthesis and gluconeogenesis played an important role in this process. SREBP-1c functions not only as a lipid regulator but also plays an important role in regulation of glucose metabolism.

## Background

Lipotoxicity plays a critical role in the pathogenesis of insulin resistance (IR) and type 2 diabetes [1]. It is widely known that experimental elevation of free fatty acid (FFA) levels leads to IR in both in vivo and in vitro studies [2–9]. It has been demonstrated that hepatic insulin resistance develops earlier than muscle insulin resistance [10–12], and the association between high levels of FFA and hepatic insulin resistance has been previously proved [13, 14]. In the liver, IR is manifested by elevated hepatic glucose production [1, 15], which is a major cause of fasting hyperglycemia in type 2 diabetes.

Hepatic lipid accumulation may be induced by 4 separate mechanisms: (1) increased hepatic uptake of circulating fatty acids, (2) increased hepatic de novo fatty acid synthesis, (3) decreased hepatic beta-oxidation and (4) decreased hepatic lipid export [16]. Uptake of circulating fatty acids is controlled by lipoprotein lipase(LPL), which is a multifunctional enzyme produced by many tissues, including adipose tissue, cardiac and skeletal muscle, islets, and macrophages, but LPL is in normally not made in the adult liver [17]. Sterol regulatory element–binding proteins (SREBPs) play a critical role in lipid synthesis. SREBPs consist of three isoforms: SREBP-1a, SREBP-1c, and SREBP-2. SREBP-1c predominates in the liver and favors the fatty acid biosynthetic pathway. SREBP-1c activates transcription of genes involved in fatty acid and triglyceride synthesis, such as the genes encoding fatty acid synthase (FAS), glyceraldehyde 3-phosphate acyltransferase (GPAT) and stearoyl-CoA desaturase (SCD) [18, 19]. Carnitine palmitoyltransferase-1 (CPT-1) plays a crucial role in fatty acid β-oxidation as a gatekeeper for entry of fatty acids into the mitochondria [20].

In addition to regulating lipid metabolism, the liver plays an important role in the regulation of gluconeogenesis. Phosphoenolpyruvate carboxylase kinase (PEPCK) and glucose-6-phosphatase (G6PC) are key gluconeogenic enzymes that catalyze one of the rate-limiting steps of gluconeogenesis and therefore contribute to increased hepatic glucose output [21, 22].

It had been demonstrated lipid levels control could reduce cardiovascular risk of dyslipidemic individuals, diet ingredients influence lipid levels [23]. We wanted to know whether inhibition of endogenous fatty acid synthesis could decrease hepatic glucose production induced by elevation of exogenous fatty acid levels. So we silenced SREBP-1c, the transcription factor controlling fatty acid synthesis, in HepG2 cells of the human hepatocellular carcinoma cell line, then we cultured these HepG2 cells in normal growth medium or treated by a high level of FFA to observe the influence of SREBP-1c silencing on glucose production in HepG2 cells as well as the expression of genes related to lipid metabolism and gluconeogenesis. This was done in order to elucidate whether a decrease in lipid synthesis could improve glucose metabolism in the liver and explore the mechanisms.

## Methods

### Cell culture

Low-passage human-derived HepG2 cells (purchased from invitrogen Carlsbad, CA, USA) were cultured in normal growth medium, Dulbecco modified Eagle’s medium (DMEM) supplemented with 4.5 mmol/L glucose, 10% fetal bovine serum, 100 U/mL penicillin, and 100 μg/mL streptomycin (all purchased from Gibco, USA) in 6-well plates. To study the influence of FFA, HepG2 cells were then cultured in normal growth medium supplemented with 0.5 mM palmitate (Sangon Biotech, Shanghai, China) in 6-well plates.

### Dissolution of palmitic acid

The 50 mmol/l fatty acid media was prepared as previously described, with slight modification [24, 25]. Briefly, palmitate was dissolved in ethanol at a final concentration of 50 mmol/l. The solution could be stored at − 20 °C. Before treatment of HepG2 cells, an appropriate amount of palmitate was incubated with 10% fatty acid-free BSA at 37 °C for 1–2 h, and an equal volume of ethanol was incubated with BSA as a control. The final concentration of BSA in the medium was 1%, the percentage of ethanol was less than 0.2% and the final concentration of palmitic acid was 0.5 mM. The approximate molar ratio of fatty acids to BSA was 10:3.

### SREBP-1c silencing

**Fig. 1:**
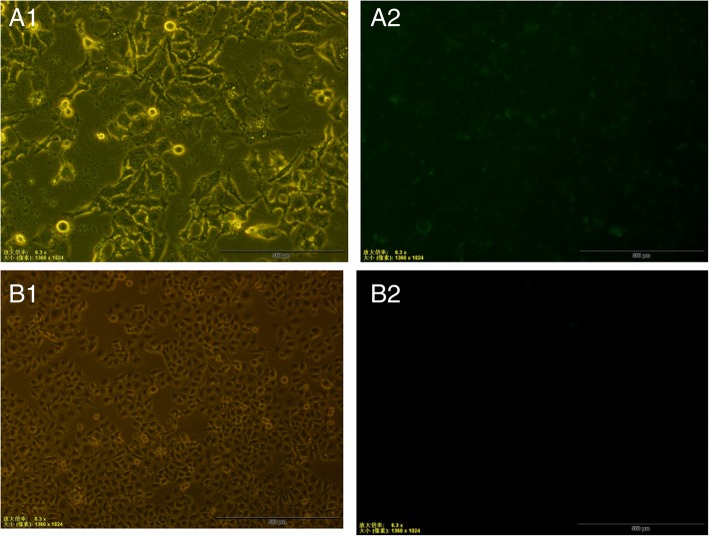
HepG2 cells transfected with scrambled siRNA (fluorescein conjugated control siRNA) (A1, Image observed under bright-field; A2, Image observed under dark-field) and HepG2 cells without treatment (B1, Image observed under bright-field; B2, Image observed under dark-field) were harvested 24 h later, and examined for fluorescein expression by fluorescent microscopy (Magnification× 6.3)**.** The transfection rate was approximately 70–80% by viewing representative nuclei

We silenced SREBP-1c in HepG2 cells using the transfection system (Santa Cruz Biotechnology, Dallas, Texas, USA) according to small interfering RNA (siRNA) transfection protocol. HepG2 cells were treated with SREBP-1c siRNA and scramble siRNA control (fluorescein conjugated control siRNA) (all purchased from Santa Cruz Biotechnology). In a 6-well tissue culture plate, 2 × 10^5^ cells per well were seeded in 2 mL antibiotic-free normal growth medium supplemented with fetal bovine serum. The cells were incubated at 37 °C in a CO_2_ incubator until the cells were 60 to 80% confluent. The following solutions were prepared: Solution A: For each transfection, 60 pmols of siRNA duplex was diluted into 100 μL siRNA transfection medium; Solution B: For each transfection, 6 μL of siRNA transfection reagent was diluted into 100 μL siRNA transfection medium. The siRNA duplex solution (solution A) was added directly to the dilute transfection reagent (solution B) using a pipette. The solution was mixed gently by pipetting the solution up and down and incubating the mixture 15 to 45 min at room temperature. The cells were washed once with 2 mL of siRNA transfection medium, the medium was aspirated and the next step implemented immediately. For each transfection, 0.8 mL siRNA transfection medium was added to each tube containing the siRNA transfection reagent mixture (solution A + solution B). The solution was mixed gently and overlaid onto the washed cells. The cells were incubated 5 to 7 h at 37 °C in a CO_2_ incubator. At the end of incubation, fluorescein conjugated control siRNA was ready to be assayed by fluorescent microscopy to ensure the success of transfection (Fig. 1). We determined the transfection rate was approximately 70–80% by viewing representative nuclei with fluorescence microscopy. Experiments were performed twice. One mL of normal growth medium containing twice the normal serum and antibiotics concentration (2× normal growth medium) was added without removing the transfection mixture. The cells were incubated for an additional 18 to 24 h. The medium was aspirated and replaced with fresh 1× normal growth medium or normal growth medium supplemented with 0.5 mM palmitate.

### Glucose production assay

**Fig. 2:**
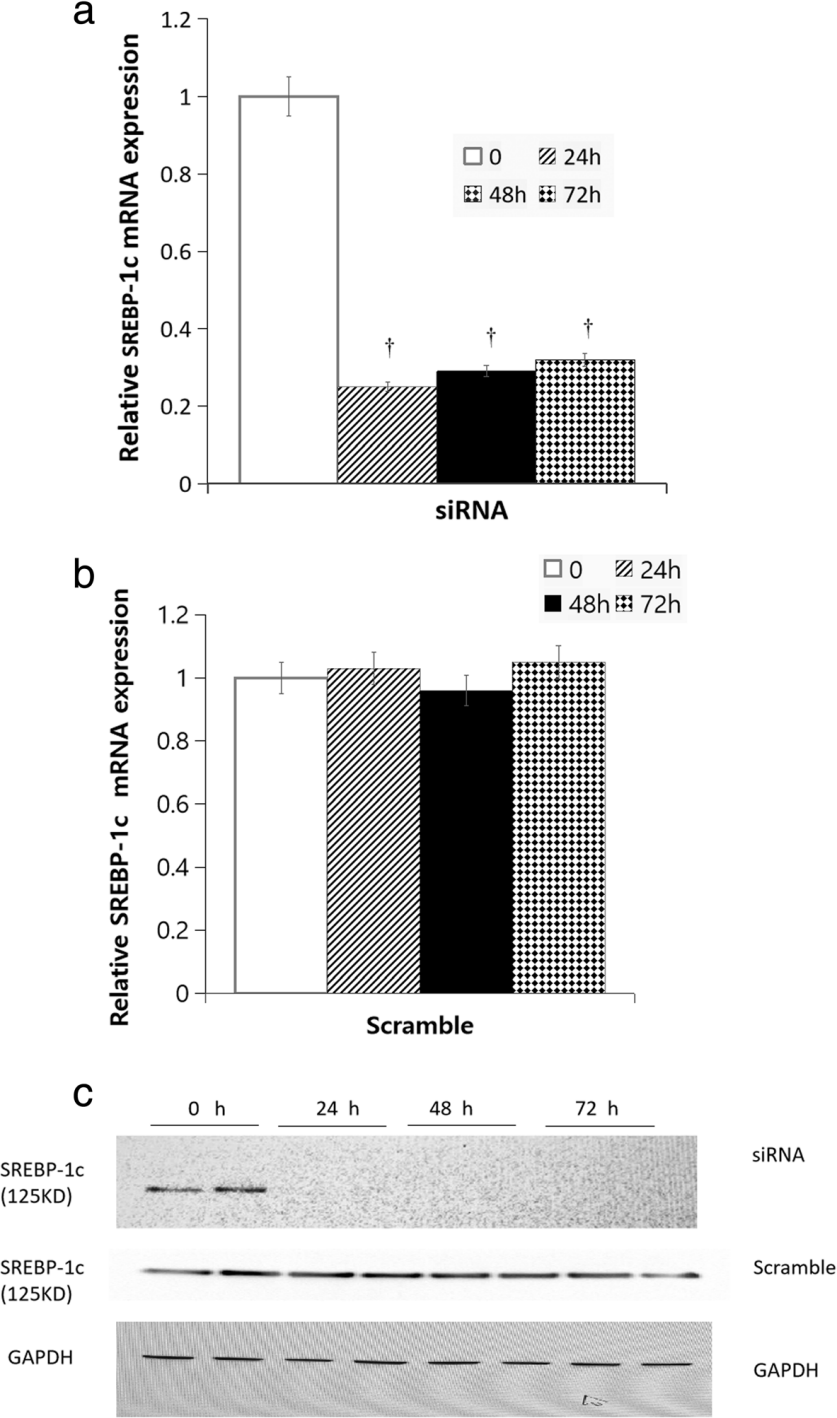
The mRNA and protein expression of SREBP-1c after SREBP-1c Silencing in HepG2 cells. **a** SREBP-1c mRNA expression in HepG2 cells treated with SREBP-1c siRNA for 24 h, 48 h, and 72 h, respectively. White squares, 0 h; wide diagonal, 24 h; black squares, 48 h; dark diamond, 72 h. **b** SREBP-1c mRNA expression in HepG2 cells treated with scramble siRNA for 24 h, 48 h, and 72 h, respectively. White squares, 0 h; wide diagonal, 24 h; black squares, 48 h; dark diamond, 72 h. **c** SREBP-1c protein expression in HepG2 cells treated with SREBP-1c siRNA and scramble siRNA for 24 h, 48 h, and 72 h, respectively. GAPDH is an internal housekeeping control. Compared with the scrambled siRNA control, SREBP-1c silencing caused the mRNA expression of SREBP-1c in HepG2 cells to decrease by approximately 75, 71 and 68% after 24 h, 48 h and 72 h, respectively. We were also able to use siRNA for 24–72 h to silence SREBP-1c protein production by more than 90% for the 125 kDa band

Glucose production was assayed after SREBP-1c silencing for 24 h, which was the period of maximal SREBP-1c silencing (Fig. 2). Cells were washed three times with phosphate-buffered saline (PBS) to remove glucose, then incubated in glucose production medium (serum-, glucose- and phenol red-free DMEM containing gluconeogenic substrates, 20 mm sodium lactate, and 2 mm sodium pyruvate). This medium was left on the cells for 10 h before being replaced with identical medium, and left overnight (10 h) for the glucose production assay [26]. A quantity of 300 μl of medium was sampled for measurement of glucose concentration using a glucose assay kit (Amplex Red Glucose Assay, Invitrogen, Carlsbad, CA, USA). Glucose concentration was normalized with cellular protein concentration.

### Western blotting

After treatment, HepG2 cells were washed with ice-cold PBS and cellular protein was collected by scraping the cells into 50 μL protein extraction buffer, after which western blotting was carried out as described [27]. Membranes were probed with the antibody against SREBP-1c (sc-365,513), protein kinase B (Akt, sc-5298), phosphorylated protein kinase B (p-Akt^s473^, sc-81,433), and glyceraldehyde-3-phosphate dehydrogenase (GAPDH) (sc-47,724) (all purchased from Santa Cruz Biotechnology), followed by incubation with horseradish peroxidase-conjugated second antibody. Immunoreactive bands were visualized by the enhanced chemiluminescence detection system.

### Quantitative real-time PCR analysis

Cells in 6-well plates were immediately washed with ice-cold PBS following treatment and lysed with 500 μL Trizol reagent (Invitrogen). Total RNA was extracted, Reverse transcription was performed with 460 ng total RNA in a total volume of 20 μl under conditions of 37 °C for 15 min, 85 °C for 5 s and 4 °C for 5 min. cDNA was synthesized using the Reverse Transcriptase kit according to the manufacturer’s protocol (PrimeScript RT Master Mix, RR036A, Takara, Biomedical Technology Co., Ltd.), the cDNA product was then amplified by real-time PCR in a total volume of 20 μl according to the manufacturer’s protocol (SYBR premix Ex Taq™ II, RR820A, Takara, Biomedical Technology Co., Ltd) using gene-specific primers (Table 1) on an ABI 7300/7500 real time PCR instrument (Applied Biosystems, Carlsbad, CA, USA). To 2 μl cDNA, 0.8 μl primer of the gene of interest and 0.8 μl primer of the reference gene were added, the reaction conditions were 40 cycles of 95 °C for 30 s, 95 °C for 5 s, 55 °C–60 °C for 34 s. Relative mRNA expression levels were calculated using the ΔΔCq method and normalized to 18S mRNA levels. Individual samples were assayed in triplicate, and the average quantification cycle (Cq) was calculated for the gene of interest and the reference gene. Based on the difference between both Cq values, the comparative was calculated. All primers were synthesized by Shanghai Sangon Biological Engineering Technology and Services Co. Ltd. (Shanghai, China). The primer efficiencies were 99.98–101.01%.

**Table 1:** Primer used for qPCR

Gene	Accession number	Nucleotide sequence(from 5′ to 3′)	Amplicon(bp)
*SREBP-1c*	NM-001005291	F: CACTGGTGGTAGATGCGGAGAA R:TCATTGATGGAGGAGCGGTAGC	139
*FAS*	NM-000043	F: AGGACATGGCTTAGAAGTGGAA, R: CTTGGTGTTGCTGGTGAGTG	166
*SCD1*	NM-005063	F: CCTGGTTTCACTTGGAGCTGTG R: TGTGGTGAAGTTGATGTGCCAGC	106
*CPT-1*	NM-001876.3	F:TGAGCACGGCAAGATGAGTC R:GAGGCAGCGATGTCTGGAAT	104
*PEPCK*	NM-002591.3	F: TGAAAGGCCTGGGGCACAT R: TTGCTTCAAGGCAAGGATCTCT	155
*G6PC*	NM-000151.3	F: TCATCTTGGTGTCCGTGATCG R: TTTATCAGGGGCACGGAAGTG	220
*18S*	NR-003286	F: CCTGGATACCGCAGCTAGGA R: GCGGCGCAATACGAATGCCCC	112

*SREBP-1c* Sterol regulatory element binding protein-1c, *FAS* Fatty acid synthase, *SCD1* Stearoyl-CoA desaturase-1, CPT-1 Carnitine palmitoyltransferase-1, *PEPCK* Phosphoenolpyruvate carboxylase kinase, *G6PC* Glucose-6-phosphatase

### Statistical analysis

Values are presented as mean ± standard deviation (SD). Statistical analysis was performed with the Statistics Package for Social Science 19 (SPSS19). The average difference of parameters between the two groups were analyzed by an independent *t* test, and differences were considered significant at *P* < 0.05.

## Results

### The mRNA and protein expression of SREBP-1c after SREBP-1c silencing in HepG2 cells

Compared with the scrambled siRNA control, SREBP-1c silencing caused the mRNA expression of SREBP-1c in HepG2 cells to decrease by approximately 75, 71 and 68% after 24 h, 48 h and 72 h, respectively (all *P* < 0.01, Fig. 2a, b). We were also able to use siRNA for 24–72 h to silence SREBP-1c protein production by more than 90% for the 125 kDa band (Fig. 2c).

### Glucose production of HepG2 cells treated by palmitate

**Fig. 3:**
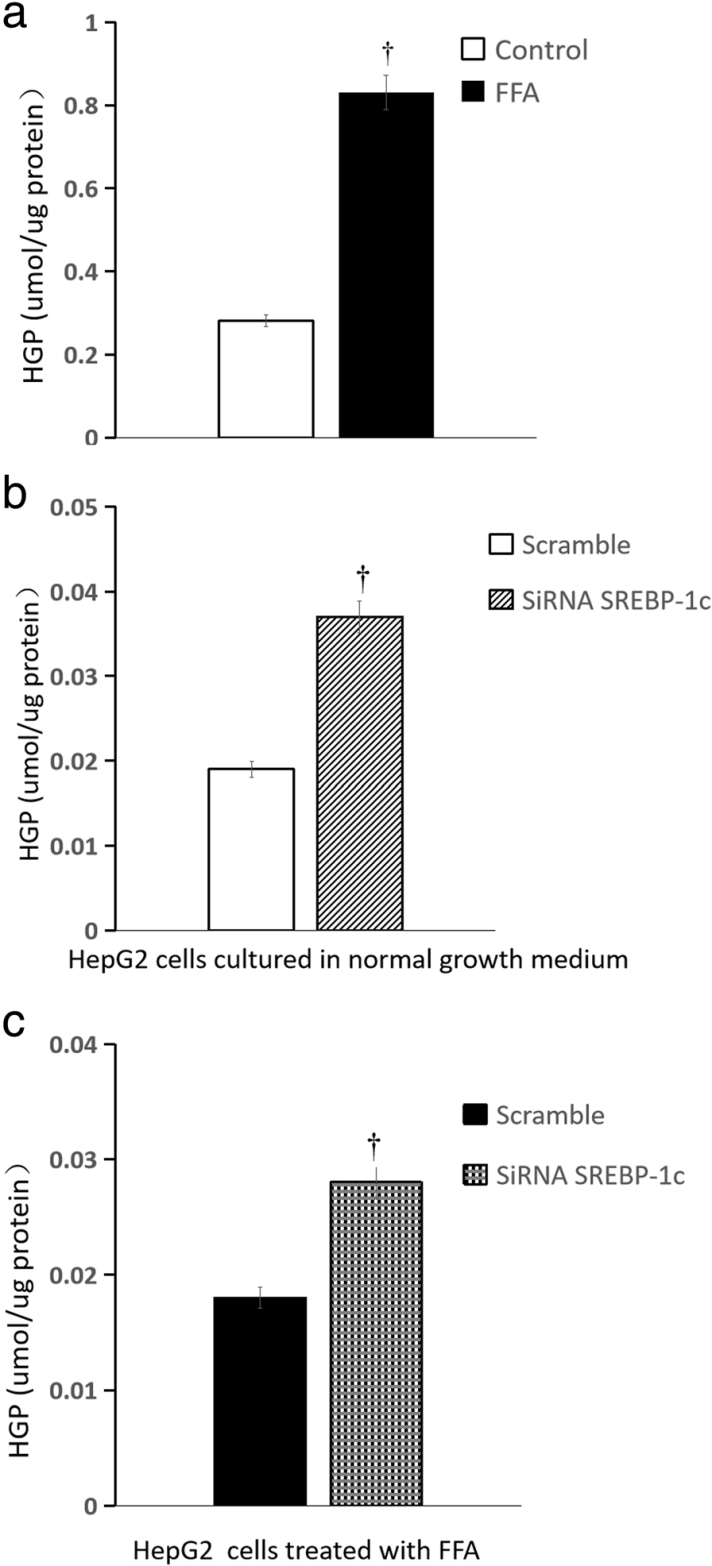
Influence of SREBP-1c silencing on glucose production in HepG2 cells. Cells were washed three times with PBS to remove glucose, then incubated in glucose production medium, the medium was left on the cells for 10 h before being replaced with identical medium, and left overnight (10 h) for the glucose production assay. **a** HepG2 cells treated by FFA (normal growth medium supplemented with 0.5 mM palmitate) vs normal growth medium (DMEM supplemented with 4.5 mmol/L glucose). **b** SREBP-1c siRNA vs scrambled siRNA in HepG2 cells cultured in normal growth medium (DMEM supplemented with 4.5 mmol/L glucose); **c** SREBP-1c siRNA vs scrambled siRNA in HepG2 cells treated by FFA (normal growth medium supplemented with 0.5 mM palmitate) (*n* = 6 wells, ^†^ *P* < 0.01 vs control). **a** white squares, normal growth medium; black square, FFA supplemented medium; **b** white squares, scrambled siRNA in HepG2 cells cultured in normal growth medium; white background on the diagonal width, SREBP-1c siRNA in HepG2 cells cultured in normal growth medium; **c** black square, scrambled siRNA in HepG2 cells treated by FFA, black scottish squares, SREBP-1c siRNA in HepG2 cells treated by FFA

Glucose production of HepG2 cells treated by palmitate was significantly increased, when compared with that cultured in normal growth medium {[(0.28 ± 0.01) vs (0.83 ± 0.02)] umol.ug^− 1^ protein, *n* = 6 wells, *P* < 0.01} (Fig. 3a).

### Influence of SREBP-1c silencing on glucose production of HepG2 cells treated by palmitate

Compared with the scrambled siRNA control, after SREBP-1c silencing, glucose production in HepG2 cells cultured in normal growth medium was significantly increased {[(0.019 ± 0.001)vs(0.038 ± 0.001)] umol.ug^− 1^ protein, *n* = 6 wells, *P* < 0.01} (Fig. 3b); SREBP-1c silencing caused glucose production in HepG2 cells treated with palmitate increased significantly {[(0.018 ± 0.001) vs(0.028 ± 0.002)] umol.ug^− 1^ protein, n = 6 wells, *P* < 0.01)} (Fig. 3c).

### Influence of palmitate on expression of genes responsible for glucose and fatty acid metabolism

**Fig. 4:**
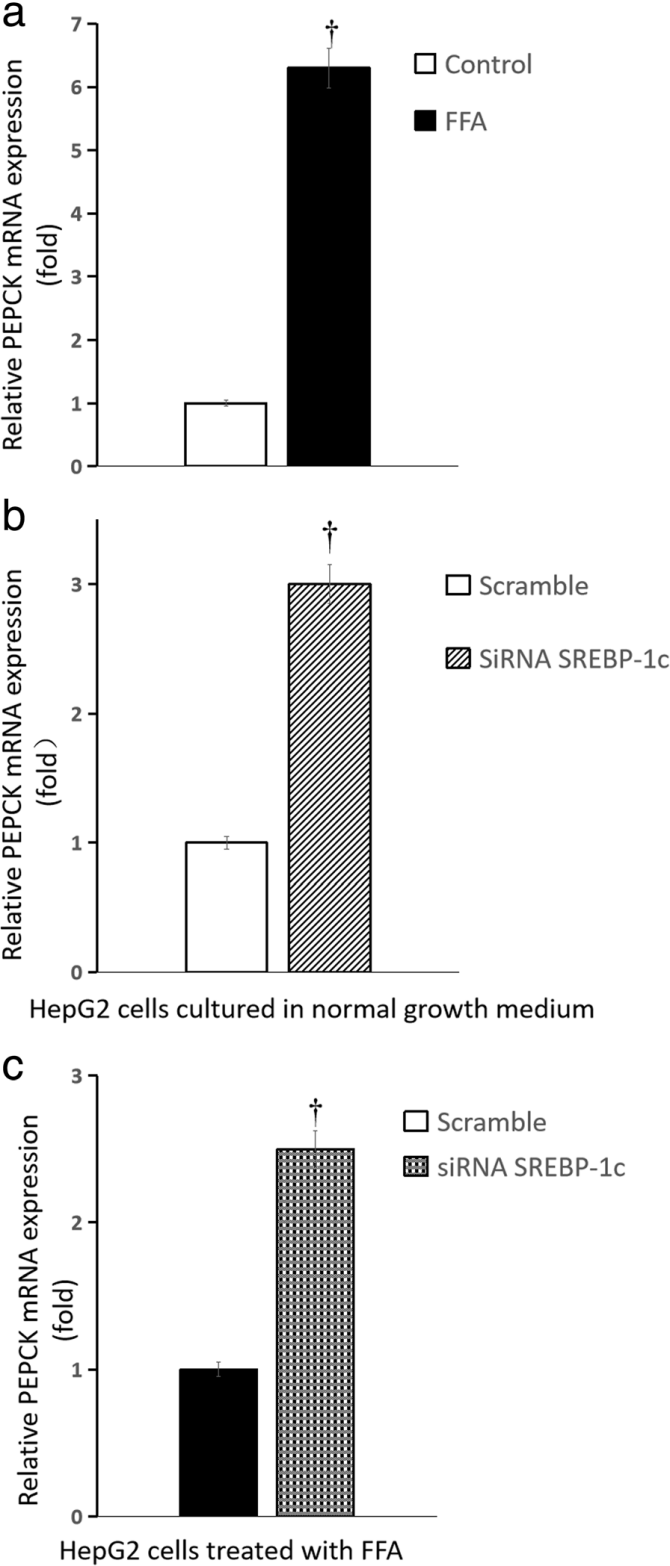
Influence of SREBP-1c silencing on PEPCK mRNA expression of HepG2 cells. **a** HepG2 cells treated by FFA (normal growth medium supplemented with 0.5 mM palmitate) vs normal growth medium (DMEM supplemented with 4.5 mmol/L glucose); **b** SREBP-1c siRNA vs scrambled siRNA in HepG2 cells cultured in normal growth medium (DMEM supplemented with 4.5 mmol/L glucose); **c** SREBP-1c siRNA vs scrambled siRNA in HepG2 cells treated by FFA (normal growth medium supplemented with 0.5 mM palmitate) (*n* = 6 wells, ^†^ *P* < 0.01 vs control). **a** white squares, normal growth medium; black square, FFA supplemented medium; **b** white squares, scrambled siRNA in HepG2 cells cultured in normal growth medium; white background on the diagonal width, SREBP-1c siRNA in HepG2 cells cultured in normal growth medium; **c** black square, scrambled siRNA in HepG2 cells treated by FFA, black scottish squares, SREBP-1c siRNA in HepG2 cells treated by FFA

**Fig. 5:**
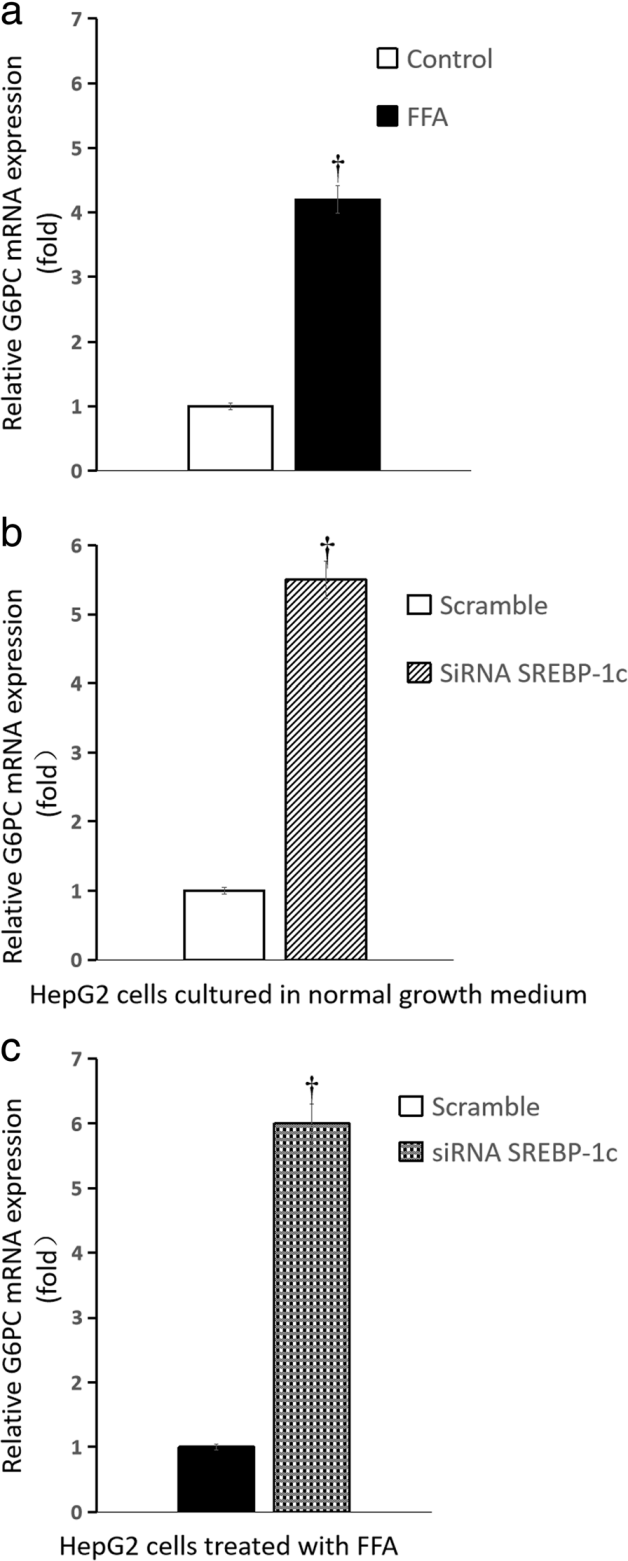
Influence of SREBP-1c silencing on G6PC mRNA expression of HepG2 cells. **a** HepG2 cells treated by FFA (normal growth medium supplemented with 0.5 mM palmitate) vs normal growth medium (DMEM supplemented with 4.5 mmol/L glucose); **b** SREBP-1c siRNA vs scrambled siRNA in HepG2 cells cultured in normal growth medium (DMEM supplemented with 4.5 mmol/L glucose); **c** SREBP-1c siRNA vs scrambled siRNA in HepG2 cells treated by FFA (normal growth medium supplemented with 0.5 mM palmitate) (n = 6 wells, ^†^ *P* < 0.01 vs control). **a** white squares, normal growth medium; black square, FFA supplemented medium; **b** white squares, scrambled siRNA in HepG2 cells cultured in normal growth medium; white background on the diagonal width, SREBP-1c siRNA in HepG2 cells cultured in normal growth medium; **c** black square, scrambled siRNA in HepG2 cells treated by FFA, black scottish squares, SREBP-1c siRNA in HepG2 cells treated by FFA

**Fig. 6:**
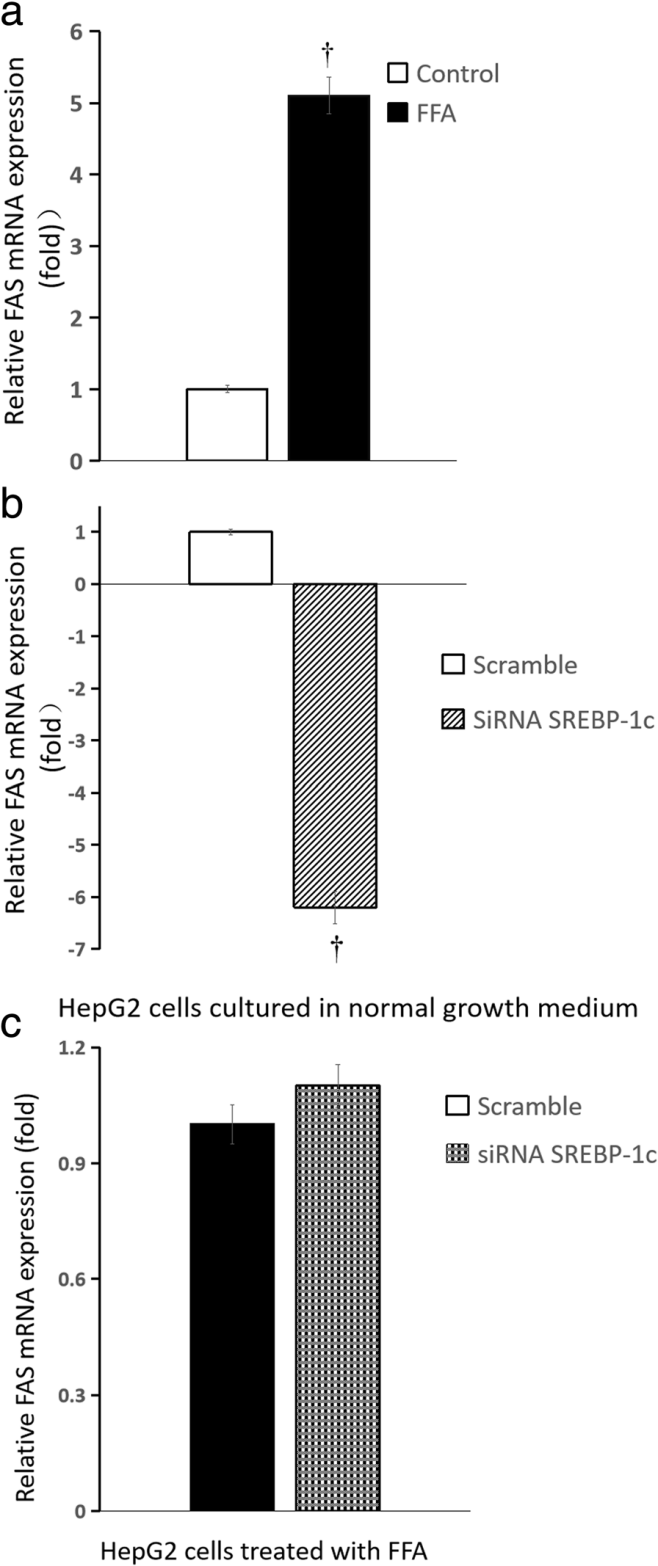
Influence of SREBP-1c silencing on FAS mRNA expression of HepG2 cells. **a** HepG2 cells treated by FFA (normal growth medium supplemented with 0.5 mM palmitate) vs normal growth medium (DMEM supplemented with 4.5 mmol/L glucose); **b** SREBP-1c siRNA vs scrambled siRNA in HepG2 cells cultured in normal growth medium (DMEM supplemented with 4.5 mmol/L glucose); **c** SREBP-1c siRNA vs scrambled siRNA in HepG2 cells treated by FFA (normal growth medium supplemented with 0.5 mM palmitate) (n = 6 wells, ^†^ *P* < 0.01 vs control). **a** white squares, normal growth medium; black square, FFA supplemented medium; **b** white squares, scrambled siRNA in HepG2 cells cultured in normal growth medium; white background on the diagonal width, SREBP-1c siRNA in HepG2 cells cultured in normal growth medium; **c** black square, scrambled siRNA in HepG2 cells treated by FFA, black scottish squares, SREBP-1c siRNA in HepG2 cells treated by FFA

**Fig. 7:**
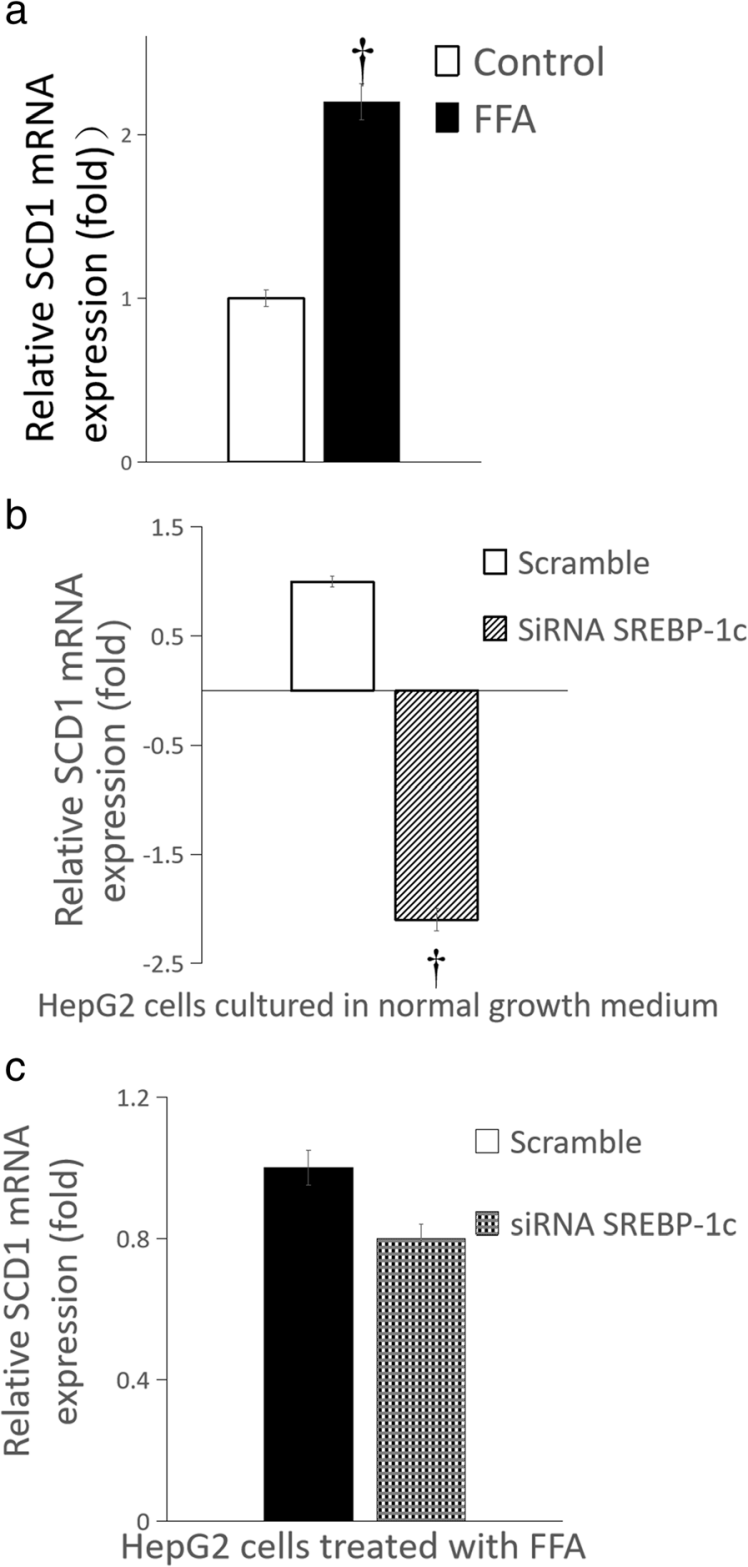
Influence of SREBP-1c silencing on SCD1 mRNA expression of HepG2 cells. **a** HepG2 cells treated by FFA (normal growth medium supplemented with 0.5 mM palmitate) vs normal growth medium (DMEM supplemented with 4.5 mmol/L glucose); **b** SREBP-1c siRNA vs scrambled siRNA in HepG2 cells cultured in normal growth medium (DMEM supplemented with 4.5 mmol/L glucose); **c** SREBP-1c siRNA vs scrambled siRNA in HepG2 cells treated by FFA (normal growth medium supplemented with 0.5 mM palmitate) (*n* = 6 wells, ^†^ *P* < 0.01 vs control). **a** white squares, normal growth medium; black square, FFA supplemented medium; **b** white squares, scrambled siRNA in HepG2 cells cultured in normal growth medium; white background on the diagonal width, SREBP-1c siRNA in HepG2 cells cultured in normal growth medium; **c** black square, scrambled siRNA in HepG2 cells treated by FFA, black scottish squares, SREBP-1c siRNA in HepG2 cells treated by FFA

**Fig. 8:**
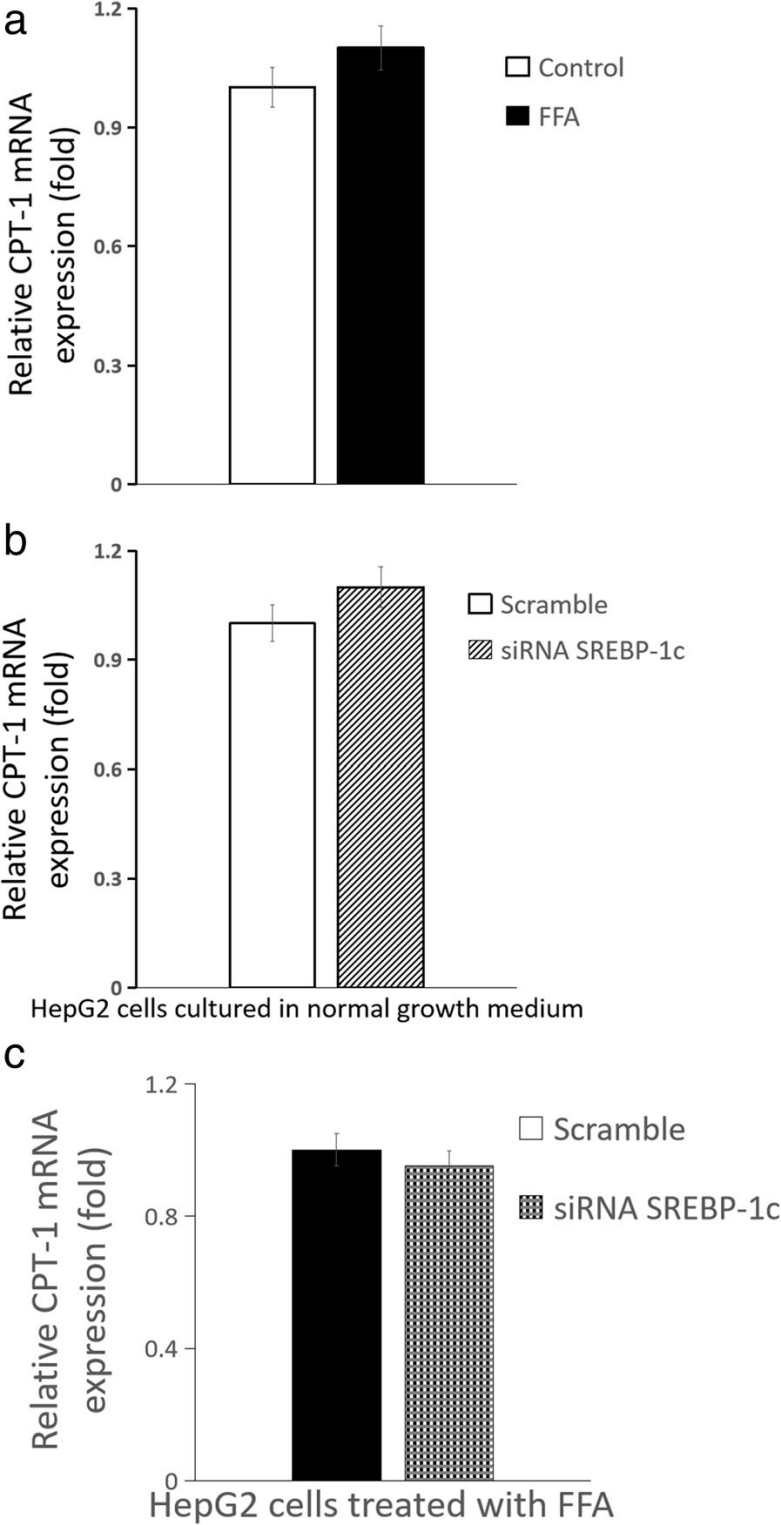
Influence of SREBP-1c silencing on CPT-1 mRNA expression of HepG2 cells. **a** HepG2 cells treated by FFA (normal growth medium supplemented with 0.5 mM palmitate) vs normal growth medium (DMEM supplemented with 4.5 mmol/L glucose); **b** SREBP-1c siRNA vs scrambled siRNA in HepG2 cells cultured in normal growth medium (DMEM supplemented with 4.5 mmol/L glucose); **c** SREBP-1c siRNA vs scrambled siRNA in HepG2 cells treated by FFA (normal growth medium supplemented with 0.5 mM palmitate) (*n* = 6 wells, † *P* < 0.01 vs control). **a** white squares, normal growth medium; black square, FFA supplemented medium; **b** white squares, scrambled siRNA in HepG2 cells cultured in normal growth medium; white background on the diagonal width, SREBP-1c siRNA in HepG2 cells cultured in normal growth medium; **c** black square, scrambled siRNA in HepG2 cells treated by FFA, black scottish squares, SREBP-1c siRNA in HepG2 cells treated by FFA

After palmitate treatment, the mRNA expression of phosphoenolpyruvate carboxylase kinase (PEPCK) and glucose-6-phosphatase (G6PC) in HepG2 cells increased by more than 5-fold and 3-fold, respectively (all *P* < 0.01) (Fig. 4a and Fig. 5a), the mRNA expression of fatty acid synthase (FAS) and stearoyl-CoA desaturase-1 (SCD1) in HepG2 cells increased by approximately 4-fold and 1.1-fold, respectively (all *P* < 0.01) (Fig. 6a and Fig. 7a), the mRNA expression of carnitine palmitoyltransferase-1(CPT-1) changed slightly (Fig. 8a) (*P >* 0.05), when compared with that cultured in normal growth medium.

### Influence of SREBP-1c silencing on expression of genes responsible for glucose and fatty acid metabolism

Compared with the scrambled siRNA control, for HepG2 cells cultured in normal growth medium, SREBP-1c silencing caused the mRNA expression of PEPCK and G6PC increased by approximately 2-fold and more than 4-fold, respectively (all *P* < 0.01) (Fig. 4b and Fig. 5b), but the mRNA expression of FAS and SCD1 decreased by approximately 6-fold and 2-fold, respectively (Fig. 6b and Fig. 7b) (all *P* < 0.01), the mRNA expression of CPT-1 changed slightly (Fig. 8b) (*P >* 0.05); for HepG2 cells treated with palmitate, SREBP-1c silencing caused the mRNA expression of PEPCK and G6PC increased by approximately 1.5-fold and 5-fold, respectively (Fig. 4c and Fig. 5c) (all *P* < 0.01), but the mRNA expression of FAS, SCD1 and CPT-1 changed slightly (Fig. 6c and Fig. 7c and Fig. 8c) (all *P* > 0.05).

### Influence of palmitate and SREBP-1c silencing on the insulin signaling pathway in HepG2 cells

**Fig. 9:**
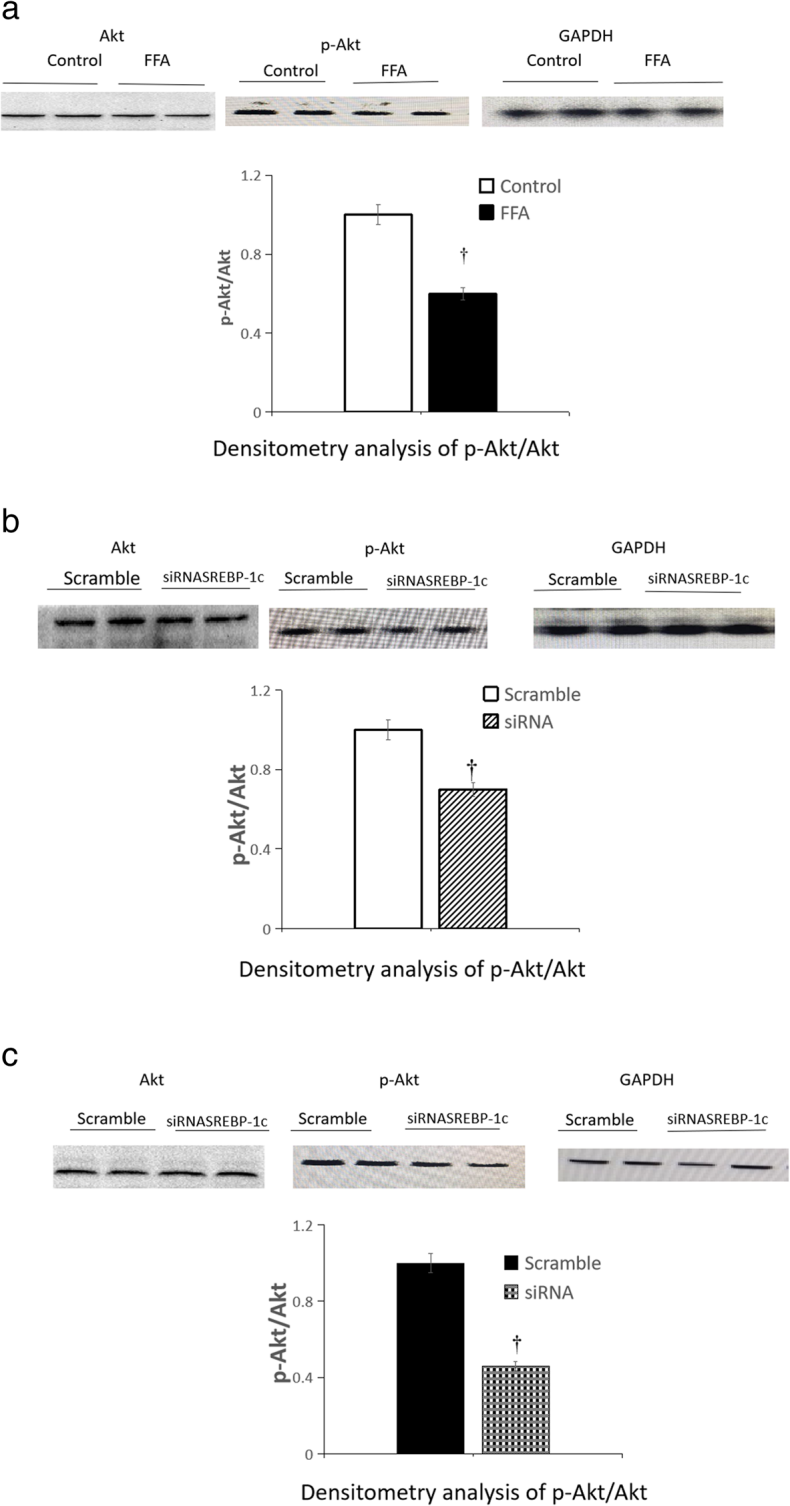
Immunoblotting of total Akt and p-Akt^s473^ in HepG2 cells in different groups. **a** Comparison of protein expression of total Akt and p-Akt ^S473^ in HepG2 cells cultured in normal growth medium and treated with FFA; **b** Influence of SREBP-1c silencing on the protein expression of total Akt and p-Akt ^S473^ in HepG2 cells cultured in normal growth medium; **c** Influence of SREBP-1c silencing on the protein expression of total Akt and p-Akt ^S473^ in HepG2 cells treated with FFA. **a** white squares, normal growth medium; black square, FFA supplemented medium; **b** white squares, scrambled siRNA in HepG2 cells cultured in normal growth medium; white background on the diagonal width, SREBP-1c siRNA in HepG2 cells cultured in normal growth medium; **c** black square, scrambled siRNA in HepG2 cells treated by FFA, black scottish squares, SREBP-1c siRNA in HepG2 cells treated by FFA. Relative level of each protein was normalized to GAPDH, an internal housekeeping control, and the control group was set to 1 (*n* = 4 wells/treatment, the data is representative of duplicate independent protein expression experiments). Values are presented as mean ± SD; †*P* < 0.01 vs control. p-Akt^s473^ is the activation of Akt, Akt proteins become phosphorylated and activated by phosphorylation of ser 473

Compared with that cultured in normal growth medium, the protein expression of p-Akt^S473^ in HepG2 cells was decreased significantly after palmitate treatment (Fig. 9a, *P* < 0.01). Compared with the scrambled siRNA control, SREBP-1c silencing decreased the expression of p-Akt^S473^ in HepG2 cells both cultured in normal growth medium and treated with a high level of FFA (Fig. 9b, c) (all *P* < 0.01).

## Discussion

In this study, we silenced the SREBP-1c gene in HepG2 cells and found the levels of SREBP-1c mRNA and protein were clearly reduced after knockdown for 24 h. This demonstrated that we silenced SREBP-1c successfully using an siRNA approach.

The liver plays a central role in the control of glucose and lipid metabolism. People with obesity are always accompanied by increased plasma FFA levels. An oversupply of FFA to the liver may affect glucose metabolism [28]. Thus, the abnormalities in hepatic glucose production in type 2 diabetic subjects could be secondary to increased FFA supply to the liver. It has been found that increased plasma FFA levels stimulate gluconeogenesis, and a correlation between hepatic glucose production and FFA levels has been demonstrated [29]. The transcription factor SREBP-1c regulates genes in the de novo lipogenesis pathway. FAS and SCD1 are the major target genes of SREBP-1c that enhance fatty acid synthesis [19], CPT-1 plays a crucial role in fatty acid β-oxidation. PEPCK and G6PC are key gluconeogenic enzymes [22].

We hypothesized inhibition of fatty acid synthesis could decrease glucose production of the liver and might be helpful to improve glucose metabolism in type 2 diabetes. It was demonstrated hepatocytes cultured in 0.5 mM palmitate for 20 h could develope IR [4] So we cultured HepG2 cells treated with 0.5 mM palmitate to mimic people with IR. Our study found that, compared with HepG2 cells cultured in normal growth medium, exposure of HepG2 cells to a high concentration of FFA for 24 h led to a significant increase in glucose production. These results are consistent with previous studies [3, 14, 30]. We further measured expression of genes responsible for fatty acid synthesis and gluconeogenesis. We found the mRNA levels of FAS and SCD-1 in HepG2 cells were significantly increased, the mRNA expression of both PEPCK and G6PC were also significantly increased, but the mRNA expression of CPT-1 changed slightly. These findings are similar to those of previous reports [31, 32], suggesting elevated FFA concentrations mainly increased the gene expression of fatty acid synthesis and gluconeogenesis which resulted in impaired lipid and glucose metabolism. It has been demonstrated that high levels of FFA facilitate de novo lipogenesis in HepG2 cells via an upregulation of SREBP-1c, which translocates into the nucleus to transcriptionally upregulate lipogenic genes including FAS and SCD1 [33], our study is in agreement with their findings. Next, we silenced the SREBP-1c gene to inhibit endogenous fatty acid synthesis in HepG2 cells. HepG2 cells were cultured in normal growth medium or treated with high levels of palmitate. In HepG2 cells cultured in normal growth medium, we found SREBP-1c deficiency decreased expression of genes responsible for lipid synthesis including FAS and SCD1, the mRNA expression of CPT-1 changed slightly. In HepG2 cells treated with FFA, the mRNA levels of FAS and SCD1 changed slightly, leading to almost the combined effects of both SREBP-1c-silenced and high levels of FFA on HepG2 cells, the mRNA expression of CPT-1 also changed slightly, suggesting SREBP-1c deficiency most likely reduced lipogenesis rather than increased fatty acid oxidation. Unexpectedly, the mRNA levels of PEPCK and G6PC in HepG2 cells both cultured in normal growth medium and treated with FFA were significantly increased after silencing of SREBP-1c. Consistent with the increase in gene expression of gluconeogenic genes, glucose production of HepG2 cells both cultured in normal growth medium and treated with 0.5 mM palmitate were significantly increased. This shows silencing SREBP-1c further increased glucose production independent of exogenous FFA levels in HepG2 cells despite inhibition of FFA synthesis. This suggests SREBP-1c is indispensable for appropriate regulation of glucose metabolism. Thus, because downregulation of lipid synthesis is at the risk of increasing glucose levels, it could not improve glucose metabolism. To our knowledge, this is the first report about glucose production of HepG2 cells after SREBP-1c silencing.

We further detected the protein expression of p-AKT ^S473^ in the insulin signaling pathway after SREBP-1c silencing. We found that compared to the scrambled siRNA control, the protein expression of p-AKT ^S473^ in HepG2 cells both cultured in normal growth medium and treated with a high level of FFA were significantly decreased. This suggested that SREBP-1c silencing induced IR in HepG2 cells. This led to increased hepatic glucose production.

## Conclusions

In conclusion, contrary to our expectations, we first found that inhibition of endogenous fatty acid synthesis by SREBP-1c silencing in HepG2 cells treated with a high level of FFA, further increased hepatic glucose production, and the genes responsible for fatty acid synthesis and gluconeogenesis played an important role in this process. Except for regulating lipid metabolism, SREBP-1c plays a very important role in the regulation of glucose metabolism. Thus, SREBP-1c is indispensable for appropriate regulation of glucose metabolism.

## Abbreviations

Akt: Protein kinase B
CPT-1: Carnitine palmitoyltransferase-1
FAS: Fatty acid synthase
FFA: Free fatty acid
G6PC: Glucose-6-phosphatase
GAPDH: Glyceraldehyde-3-phosphate dehydrogenase
GPAT: Glyceraldehyde 3-phosphate acyltransferase
HGP: Hepatic glucose production
IR: Insulin resistance
p-Akt ^S473^: Phosphorylated Protein kinase B
PEPCK: Phosphoenolpyruvate carboxylase kinase
SCD1: Stearoyl-CoA desaturase-1
siRNA: Small interfering RNA
SREBP-1c: Sterol regulatory element binding protein-1c
